# Effect of Duration of Exposure to Males on Female Reproductive Performance of the Green Lacewing, *Chrysoperla agilis* (Neuroptera: Chrysopidae)

**DOI:** 10.3390/insects12060560

**Published:** 2021-06-18

**Authors:** Konstantinos Athanasiadis, Maria L. Pappas, George D. Broufas

**Affiliations:** Department of Agricultural Development, Democritus University of Thrace, Pantazidou 193, 68200 Orestiada, Greece; athanasiadiskostis@gmail.com (K.A.); gbroufas@agro.duth.gr (G.D.B.)

**Keywords:** biological control, *Chrysoperla* spp., green lacewings, mating, predators

## Abstract

**Simple Summary:**

The effect of duration of exposure to males on female longevity and egg production of the predatory insect *Chrysoperla agilis* Henry et al. was studied under laboratory conditions. Newly emerged adult females of *C. agilis* were placed in cages alone or with males, the latter either for 1 week or for the entire females’ lifetime. Females in continuous presence of males laid considerably more eggs than females that had access to males for 1 week. Virgin females lived the longest, and those in the presence of males lived the shortest. Egg hatchability and offspring sex ratio were similar for females exposed to males, irrespective of the duration of exposure. We showed that the presence of one male for 1 week early in the adult life of a female is not sufficient for *C. agilis* reaching maximum reproduction. Our results are applicable in mass-rearing of *C. agilis* to be used in biological control against agricultural pests.

**Abstract:**

*Chrysoperla agilis* Henry et al. is one of the five cryptic species of the *carnea* group found in Europe. They are known to widely occur in agricultural fields and survive and reproduce in a wide range of temperatures. The reproductive biology of the cryptic species is poorly known, especially regarding the number of matings required for the females’ maximum reproductive output. We recorded the egg production and longevity of virgin females, as well as of females that had access to males for 1 week or for their lifetime. Longevity of *C. agilis* females with access to males was similar whether these were present for 1 week or for their lifetime (64.8 and 66.1 days, respectively). On the other hand, oviposition was higher in the long-term exposure to males (302.1 vs. 421.1 eggs, respectively). Virgin females lived longer (94.1 days) than mated females and laid a low number (54.5) of (unfertile) eggs. Egg hatchability and progeny sex ratio were similar in treatments with males. Nevertheless, the highest value (0.1321) of intrinsic rate of increase (*r_m_*) was recorded when females were continuously exposed to males. These results are relevant to biological control and could be applicable in mass-rearing *C. agilis* and predicting its population dynamics in the field.

## 1. Introduction

Lacewings of the family Chrysopidae are polyphagous predators of aphids and other soft-bodied arthropods. Certain chrysopid species, mainly those of the genus *Chrysoperla*, are important biological control agents of key herbivorous pests and have been successfully used in biological control programs in greenhouses and in the field [1,2].

Among chrysopids, the *Chrysoperla carnea* (Neuroptera: Chrysopidae) group includes at least 16 species of green lacewings that can be distinguished by their unique species-specific substrate-borne courtship songs [3,4]. They are known to comprise a complex species. Generally referred to as *Chrysoperla carnea* (Stephens) sensu lato [2,5], consisting of reproductively isolated species that have no morphological differences [6], the species produce courtship songs of low frequency by vibrating their abdomen on the substrate when they are ready to mate. Their songs are a prerequisite for the mating to occur [5,7]. *Chrysoperla carnea* s.l. is the most commonly used species in augmentative biological control.

Five of the species of the *C. carnea* group, namely *C. agilis* Henry et al., *C. carnea* sensu stricto (Stephens), *C. lucasina* (Lacroix), *C. mediterranea* (Hölzel), and *C. pallida* Henry et al., are all found in Europe with overlapping geographical distribution [7]. *Chrysoperla agilis* in particular is a valid biological species that can be distinguished from the other cryptic species by the distinct mating signals of females and males [7]. It has been recorded in the southern parts of Europe, the Mediterranean islands and from southwestern Asia to northern Iran, as well as in central Alaska [4,8].

The wide distribution and habitat range of *C. agilis* combined with its broad feeding habits and ability to develop and reproduce in a wide range of temperatures [9] render it a promising biological control agent in various crops against several pests [8]. Moreover, the abundance of *C. agilis* in agricultural fields [8,10] suggests its role as a biological control agent within its distribution ranges. Hence, *C. agilis* seems to bear certain ecological traits that render it suitable for release in specific agroecosystems where other cryptic species such as *C. lucasina* or *C. mediterranea* might prove completely ineffective [8]. In Greece, it has been recorded in fruit trees, vegetables, and field crops feeding on aphids, spider mites, and other soft-bodied arthropods (Pappas et al., unpublished data).

As with other species of the *carnea* group, *C. agilis* can be easily reared in the lab; larvae can feed and develop successfully on foods such as *Ephestia kuehniella* (Lepidoptera: Pyralidae) eggs, whereas adults are not predaceous and can be fed on protein-based liquid diets [2,9]. In accordance, it can be suggested that the same techniques applied for mass-rearing *C. carnea* s.l. could be adapted for the commercialization and use of *C. agilis* in biological control against key arthropod pests (e.g., aphids and mealybugs) in the area of its expansion. Sex ratio and number of required matings for *C. agilis* to reach the maximum reproductive output of females may be key determinants of the success of a mass-rearing system. Despite current knowledge on the mating and oviposition behavior of several chrysopid species (*C. carnea* s.l. included) [11,12,13], relatively little is known about the *carnea*-group cryptic species in this regard.

*Chrysoperla agilis* is a promising candidate for use in biological control in the areas of its natural occurrence. We herein assessed the impact of the duration the females had access to males on the survival, egg production, and progeny sex ratio of *C. agilis*. Furthermore, we calculated the intrinsic rates of *C. agilis* population increase in the different treatments. We hypothesized that multiple matings may be required for *C. agilis* females to reach their maximum reproductive output.

## 2. Materials and Methods

### 2.1. Chrysoperla agilis Laboratory Rearing

The laboratory colony of the lacewing *C. agilis* was established with individuals collected in cotton crops by hand net in 2015 from the area of Orestiada (northern Greece). Experiments were performed with adults deriving from the F3 progeny of the field-collected individuals. Adults were paired and maintained in cylindrical plastic cages (35 cm in height × Ø 25 cm) at 25 °C, 65–75% RH, and a photoperiod of 16:8 h (L:D), as described by Pappas et al. [14]. Briefly, adults were reared in groups of females and males in cages and were fed with a protein-based liquid food (a volumetric mixture of honey, sugar, yeast hydrolysate, and water, 1:1:1:1) that was applied daily on the top of a mesh that covered each cage. Chrysopid larvae were reared on *E. kuehniella* eggs until pupation. Lacewing species was identified with both song analysis and morphological traits by Prof. Charles S. Henry (University of Connecticut).

### 2.2. Experimental Setup

The goals of our experiments were to assess the impact of duration of exposure to males on the performance of *C. agilis* females, as well as its impact on the predator’s intrinsic rate of population increase. Toward this end, our study consisted of two phases. During the first phase (juvenile development), we reared lacewing individuals until adulthood to obtain the experimental females and to calculate the mean developmental time and survival during development of *C. agilis* in the experimental conditions. In the second phase, these young females were split into three groups, each representing one experimental treatment, i.e., (1) virgin females, (2) females with males for 1 week, and (3) females with males for their lifetime. During the second phase, we were interested to assess the impact of duration of exposure to males on the egg production and longevity of females. Eventually, combining data from both experimental phases, we were able to calculate intrinsic rate of population increase (*r*) values for treatments (2) and (3). Below, we describe experimental procedures during each phase.

### 2.3. Developmental Time and Survival during Juvenile Development

Within 12 h of laying, eggs were collected from the lab colony and transferred individually to plastic Petri dishes (Ø5 cm) at 25 °C, 65–75% RH, and 16:8 h (L:D). Frozen UV-sterilized eggs of the flour moth *E. kuehniella* were provided *ad libitum* in each Petri dish as food for the lacewing larvae. To ensure proper ventilation, an opening approximately 3 cm in diameter was cut on the lid cover of each Petri dish and covered with a thin fabric to prevent lacewing larvae from escaping.

Twice daily, at 12 h intervals, the Petri dishes were inspected, and the survival and developmental stage of the lacewing larvae were recorded until adult emergence. During inspection, the presence of exuvium was used as evidence for successful molting and change from one instar to the next. The presence of a visible black disc in the cocoon was used as the only morphological evidence for the successful molting of prepupal to the pupal stage [2,15]. The estimated values of the juvenile developmental time (22.6 days) and the percentage of emerging adults were used in the estimation of respective values of the intrinsic rate of increase (*r_m_*).

### 2.4. Female Longevity and Reproduction

Emerging adults were sexed according to the morphology of their genitalia [16], allocated in three groups and transferred individually to cylindrical plastic cages (350 mL in volume) similar to those described by Pappas et al. [14]. Briefly, a hole was opened at the bottom of each cage to provide water to the insects via a cotton wick that was placed in contact with water in a Petri dish (Ø5 cm). Furthermore, the lid of each cage was covered with a fine mesh that allowed for air circulation in the cage. Each group consisted of 15 young (1 day old) females which, depending on the treatment: (1) remained virgin throughout their life, (2) shared the cage with one male for 1 week, or (3) shared the cage with one male for their lifetime. In the latter treatment, young males from the lab colony were replaced once per week in each cage to control for the possible effects of male age on the female reproductive output. The same protein-based liquid food used for the lab colony was also provided to the experimental adult lacewings. Water access was provided through a cotton wick that was always in contact with water in the cage. Every day, all cages were inspected, and survival of the females and egg production (i.e., the number of eggs laid on the paper covering the inside of the cage and on the mesh) were recorded throughout their lifespan. In all treatments, survival and fecundity of females were recorded every 24 h until their death.

### 2.5. Intrinsic Rate of Population Increase

Life and fertility table parameters were constructed by combining data from the juvenile development and adult survival and egg production, as described by Wang and Tsai [17]. The intrinsic rates (*r_m_*) of population increase (i.e., per capita change in the population per unit time) were estimated by iteratively solving the Euler–Lotka equation, as described by Birch [18].

$$\overset{n}{\underset{\begin{array}{c} x=0 \end{array}}{\sum }}e^{-r_{m}x}l_{x}m_{x}=1,$$

where *x* is the mean age class, *m_x_* is the mean number of female progeny per female of age *x*, and *l_x_* is the probability of survival to age *x*.

### 2.6. Egg Hatchability and Sex Ratio

Egg hatchability was estimated by collecting 40 eggs (2–4 eggs per female) per treatment at three different time points of females’ lifetime (15, 25, and 45 days). The eggs were transferred individually in Eppendorf tubes, maintained in the same conditions as the parental pairs, and inspected daily for a period of 1 week. The number of newly hatched larvae was recorded, and the percentages of egg hatchability were estimated. Subsequently, the newly hatched larvae were individually transferred in Petri dishes and reared until adult stage on frozen UV-sterilized eggs of *E. kuehniella.* The emerging adults were sexed, and sex ratios of the offspring for the different treatments were calculated.

### 2.7. Statistical Analysis

Females’ longevity and mean total oviposition per female were compared with the nonparametric Kruskal–Wallis test followed by all possible pairwise comparisons among the different treatments with the nonparametric Mann–Whitney *U* test. The Kaplan–Meier method with the log rank test was used to compare the survival curves of the adult females among the different treatments, whereas egg hatching and sex ratio percentages of the progeny among treatments were compared with the *χ*^2^ test. A linear mixed model with treatment and female age as fixed factors and female individual as random factor was used to evaluate the effect of treatment and age on egg production. Means and standard error values of the *r_m_* at the different treatments were estimated with the bootstrap technique (1000 bootstrap samples), and means were subsequently compared with the *t*-test. For all tests, significance levels were α = 0.05. All statistical analyses were performed using SPSS [19].

## 3. Results

### 3.1. Effects of Duration of Exposure to Males on Egg Production and Female Longevity

**Figure 1:**
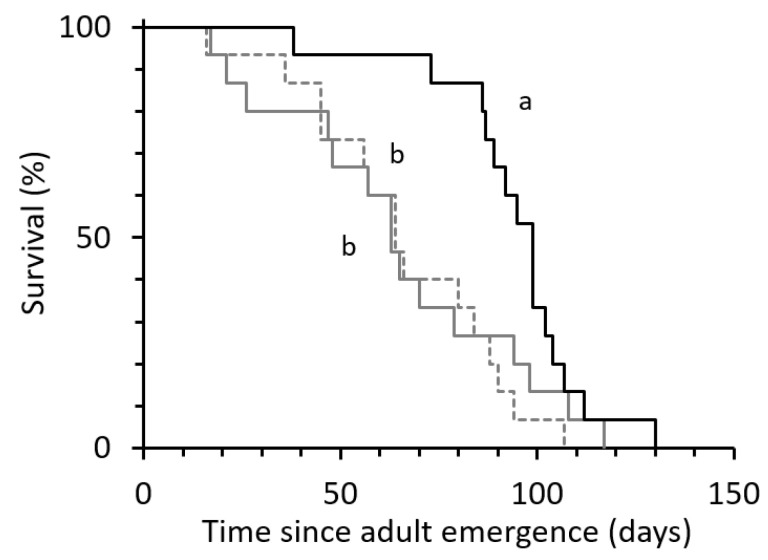
Kaplan–Meier survival curves of *Chrysoperla agilis* females, depending on the duration of male availability to females for mating: (1) Virgin females (compact black line, *n* = 15); (2) 1 week mating: the male present for 1 week (gray line, *n* = 15); (3) lifetime: male present for the female’s lifetime (dotted gray line, *n* = 15). Different letters on each curve indicate significant differences between treatments (log rank test, *p* < 0.05).

**Figure 2:**
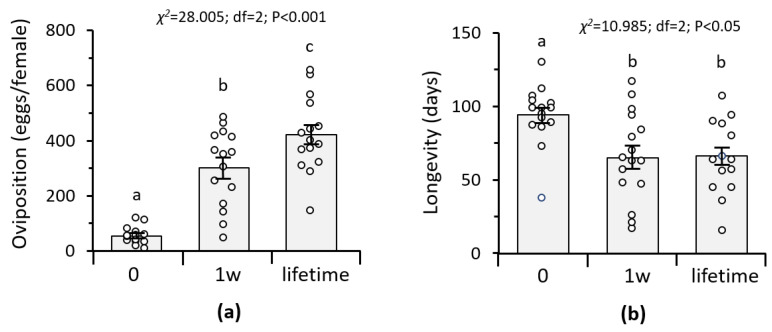
Mean total oviposition (**a**) and longevity (**b**) of *Chrysoperla agilis* females, depending on the duration of male availability to females for mating: (1) 0: virgin females (*n* = 15); (2) 1w: male present for 1 week (*n* = 15); (3) lifetime: male present for the female’s lifetime (*n* = 15). Different letters indicate significant differences between treatments using Kruskal–Wallis test followed by Mann–Whitney U-test for all pairwise comparisons, *p* < 0.05.

To assess the impact of duration of exposure to males on egg production and longevity of *C. agilis*, we exposed young females to males for variable durations (1 week, lifetime) or not (virgin females). Hence, we did not observe or count the number of successful matings but hypothesized that the different treatments were adjusted as follows: (1) virgin females: no exposure of females to males; (2) 1 week mating: females were exposed to males for 1 week in the same cage; (3) lifetime: females were exposed to males for the female’s lifetime. There was a significant effect of treatment (i.e., duration of female exposure to males) on female survival rate (*χ*^2^ = 8.191; d.f. = 32; *p* = 0.017). Virgin females lived on average 94 days, significantly longer than females exposed to males (*χ*^2^ = 10.985; df = 2; *p* < 0.001, Figure 1 and Figure 2). Longevity of females that had access to males throughout their lifetime did not differ significantly from the longevity of females that were exposed to males for a shorter period, i.e., 1 week (*χ*^2^ = 0.286; *p* = 0.593, Figure 1).

Virgin females did lay a low number of eggs (approximately 54 eggs on average) as compared to females exposed to males for variable time periods (approximately 302 to 421 eggs/female). Lifetime oviposition of females continually exposed to males was significantly higher compared to the females exposed to males for only 1 week (Figure 2).

**Figure 3:**
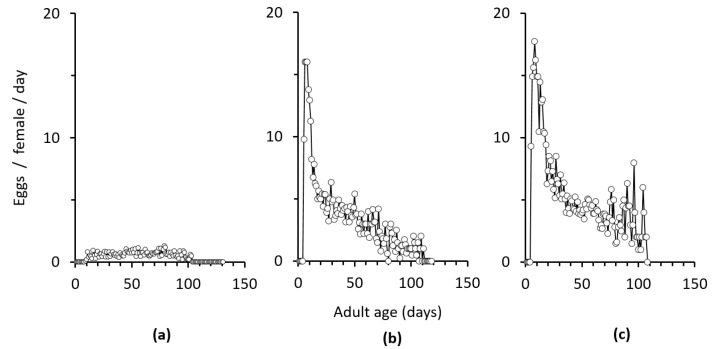
Mean daily oviposition of *Chrysoperla agilis* females depending on the duration of male availability to females for mating: (**a**) virgin females (*n* = 15); (**b**) 1 week mating: the male present for 1 week (*n* = 15); (**c**) lifetime: male present for the female’s lifetime (*n* = 15).

There was a significant effect of the interaction between treatment and female age on egg production (F = 11.403, df = 222, *p* < 0.001). The number of eggs laid by females differed significantly among treatments (F = 45.83, df = 2, *p* < 0.001) and with female age (F = 29.41, df = 131, *p* < 0.001). Daily oviposition was significantly higher when females were continuously exposed to males as compared to the 1 week exposure (Figure 3). The number of eggs laid by virgin females was low and varied from approximately 0.3 to 1.3 eggs per female per day for more than 60% of their lifetime (Figure 3). In contrast, egg production of the females that were exposed to males increased during the first 10 days of the oviposition period and then ceased gradually (Figure 3). Females that had access to males laid eggs for approximately 90–100 days, whereas post-oviposition lifespan was very short compared to virgin females (Figure 3).

### 3.2. Egg Hatchability

**Figure 4:**
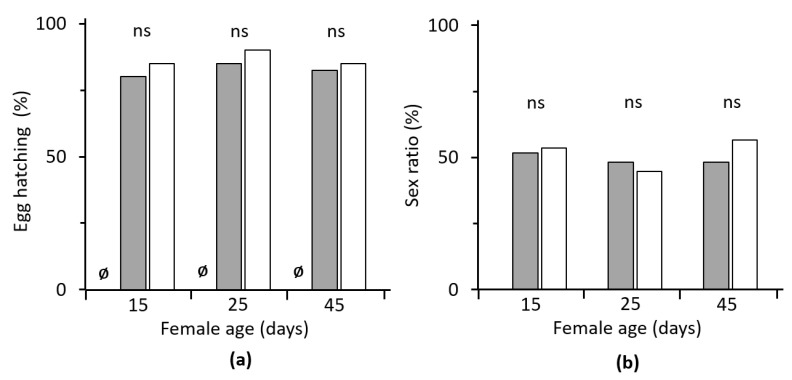
(**a**) Hatchability of *Chrysoperla agilis* eggs laid by females of different ages (*n* = 40) and (**b**) offspring sex ratio. Depending on the duration of male availability to females for mating, eggs were obtained by females that had the option of (**a**) 1 week mating: the male present for one week (gray bars, *n* = 29–31), or (**b**) lifetime: male present for the female’s lifetime (white bars, *n* = 28–30); *χ*^2^-test: ‘ns’ denotes no significant differences, and Ø denotes zero egg hatching for virgin females.

The hatchability of eggs laid by *C. agilis* females of different age ranged from 80% to 90% and was not significantly affected by treatment, i.e., duration of female exposure to males (0.092 ≤ *χ*^2^ ≤ 0.949; *p* > 0.05). Eggs laid by virgin females did not hatch (Figure 4a).

### 3.3. Progeny Sex Ratio

The estimated values of sex ratio in the progeny of *C. agilis* females of the different treatments, i.e., 1 week vs. lifetime exposed to males, ranged from 44.8% to 52.6% (on average, 49.4% and 51.7%, respectively) and were not significantly affected by the treatment in the three different age classes (0.023 ≤ *χ*^2^ ≤ 0.416, *p* > 0.05) (Figure 4b).

### 3.4. Intrinsic Rate of Population Increase

The estimated values for intrinsic rate of natural increase (*r_m_*) of *C. agilis* ranged from 0.1237 to 0.1321, with increased duration of female exposure to males resulting in a significantly higher *r_m_* value (Table 1).

## 4. Discussion

In the present study, we assessed the impact of continuous presence of males versus 1 week presence or no male presence on the reproductive performance of *C. agilis* females. We found that virgin females lived longer than mated females; hence, we may assume that mating and subsequent oviposition are energetically costly. Exposure to males can also be costly through repeated mating attempts, disruption of feeding, toxicity of seminal fluid molecules, transfer of sexually transmitted pathogens, and damage to the female reproductive tract during mating [20,21]. Whether any of the above is relevant to our study species requires further investigation. The continuous presence of males was shown not to affect the longevity of females, progeny sex ratio, or egg hatchability. Nevertheless, exposing females continuously to males resulted in increased egg production by the females. In addition, the values of intrinsic rate of population increase (*r_m_*) were higher when *C. agilis* females had access to males continuously during their lifetime as compared to 1 week exposure.

In chrysopids, physiological sexual maturation is triggered by mating, which acts by further stimulating oviposition [22,23]. Nevertheless, egg laying by virgin females is not uncommon and, although relatively weak, it has been reported for various chrysopids such as *Chrysopa oculata*, *Chrysopa perla*, and *C. carnea* [22]. This was also shown in the present study, with *C. agilis* females laying a low number of eggs for a relatively high number of days during their lifetime. Egg laying by unmated chrysopid females is known to relate to egg cannibalism by adults of both predatory and nonpredatory chrysopid species, especially in laboratory rearing and when females are fed with suboptimal diets [15]. However, for egg cannibalism to happen, females should extract their own eggs from their genital opening with the help of their mandibles. Moreover, it should relate to the need of the females to compensate for poor diets [15]. In our study, eggs laid by virgin females were mostly stalked and were found either on the walls or attached on the cloth covering each cage. Whether these eggs could be used as adult food, as well as the adaptive significance of this behaviour of *C. agilis* virgin females, needs to be further studied.

Females of the majority of insects maximize lifetime offspring production through multiple mating [20]. Our results suggest the possibility for more than one mating to occur when males were continuously present for the females’ lifetime. Other studies have shown that just one mating suffices to sustain oviposition for a lifetime [11,12,22,24,25,26]. Nevertheless, there is also evidence for remating in certain chrysopid species, among them *Chrysoperla* spp., which were shown to remate after long oviposition periods, as well as *Ceraeochrysa* spp. [11,13,25,27]. Specifically, in *Chrysoperla* sp., egg-laying females will only re-mate when their egg productivity is about to decline; hence, most females are generally expected to mate fewer than five times in total before senescing, whereas males are capable of remating at least once a day [12]. In accordance, a second mating can occur in *C. carnea* [27]. Further studies are needed to assess whether the other cryptic species of the *carnea* group require one or multiple matings to reach their maximum reproductive output. Additionally, since we did not record copulations in our experiments, future studies should be performed to understand the exact number of matings occurring until the death of *C. agilis* females.

The need to remate can provide a periodic stimulus for oviposition [28,29] or, when stored sperm is nearly depleted, enable chrysopids to regain sexual receptivity after copulation [11]. In the latter case, re-mating with different males in the field would also increase the genetic variability of the offspring from each individual female [30,31]. On the other hand, the need to remate also exerts cost(s) such as low fecundity risk when males are scarce [32] and increased exposure to natural enemies [13]. In the present study, we recorded a significant increase in the longevity of virgin females as compared to mated females, suggesting the existence of energetic costs in the process of mating *per se*. Furthermore, the calculated *r_m_* values of population increase in *C. agilis* were found to be significantly different when males were constantly present with females or for 1 week only. Our results suggested that exposure to males beyond 1 week is crucial for maintaining high rates of fecundity in this species. The requirements of continuous presence of males in mass-rearing of other species (e.g., *C. cincta*) or the release of high numbers of males of the latter species in the field [13] may also be related to the rearing and release of *C. agilis*. This suggests that the production and release of *C. agilis* should also include these cost parameters for their mass production.

Overall, *C. agilis* is a widespread species in the area of its expansion and a promising candidate for mass-rearing and use in augmentative biological control. Ease of rearing with the same methods used to rear *C. carnea*, high survival, fast development and high reproductive potential in a wide range of temperatures, and occurrence in agricultural fields [9], as well as high egg hatchability percentage and a balanced progeny sex ratio, are key traits that render *C. agilis* a promising biological control agent. In this study, we found that exposing females for 1 week should suffice for reaching approximately 70% of the maximum reproduction, whereas continuous exposure to males is necessary for reaching the highest innate capacity for population increase of *C. agilis*. This information is applicable for mass-rearing and releasing *C. agilis* in the field. Our results could be applicable in optimizing mass-rearing systems, as well as for assessing the ecological costs of continued exposure to males in chrysopids and understanding the population dynamics in the field of one of the most commonly found cryptic species of the *carnea* group.

To optimize their use in biological pest control, future studies should focus on species-specific differences in the reproductive biology among cryptic species of the *carnea* group.

## Figures and Tables

**Table 1:** Intrinsic rates of population increase (*r_m_*) of *Chrysoperla agilis* depending on the duration of male availability to females for mating.

Treatments	*r_m_* (day^−1^)
Virgin females	-
1 week mating	0.1237 ± 0.0004a ^1^
Lifetime	0.1321 ± 0.0001b

^1^ Values followed by different letters are significantly different (*t*-test: *t* = 19.76; *p* = 0.001).

## Data Availability

The datasets generated and analyzed during the current study are available from the corresponding author upon reasonable request.
